# Intrapulmonary autologous transplant of bone marrow-derived mesenchymal stromal cells improves lipopolysaccharide-induced acute respiratory distress syndrome in rabbit

**DOI:** 10.1186/s13054-018-2272-x

**Published:** 2018-12-20

**Authors:** Mohammad Reza Mokhber Dezfouli, Massoumeh Jabbari Fakhr, Sirous Sadeghian Chaleshtori, Mohammad Mehdi Dehghan, Alireza Vajhi, Roshanak Mokhtari

**Affiliations:** 1 Department of Internal Medicine, Faculty of Veterinary Medicine, University of Tehran, Tehran, Iran; 2 Department of Surgery and Radiology, Faculty of Veterinary Medicine, University of Tehran, Tehran, Iran; 3 Institute of Biomedical Research, University of Tehran, Tehran, Iran

**Keywords:** ARDS, BM-MSCs, Stromal cell therapy, Intrapulmonary, Rabbit

## Abstract

**Background:**

Lung diseases such as acute respiratory distress syndrome (ARDS) have a high incidence worldwide. The current drug therapies for ARDS have supportive effects, making them inefficient. New methods such as stromal cell therapy are needed for this problem.

**Methods:**

This research was performed with ten New Zealand rabbits in two groups. Bone marrow aspiration was performed on the treated group, and mesenchymal stem cells were isolated and cultured. The experimental model of ARDS was induced using LPS from *Escherichia coli* strain O55:B5. Then, 10^10^ bone marrow mesenchymal stem cells (BM-MSCs) were autologously transplanted intrapulmonary in the treatment group, and 1–2 ml of PBS in the control group. The clinical signs, computed tomographic (CT) scans, echocardiography, blood gas analysis, complete blood count, and cytokine levels were measured before and at 3, 6, 12, 24, 48, 72, and 168 h after BM-MSC transplant. Finally, the rabbits were killed, and histopathological examination was performed.

**Results:**

The results showed that BM-MSCs decreased the severity of clinical symptoms, the number of white blood cells and heterophils in the blood, the total cell count, and number of heterophils and macrophages in bronchoalveolar lavage, and balanced the values of arterial blood gases (increase in partial pressure of oxygen and O_2_ saturation and decrease in the partial pressure of carbon dioxide). They also downregulated the tumor necrosis factor (TNF)-α and interleukin (IL)-6 concentrations and increased the IL-10 concentrations at different times compared with time 0 and in the control group, significantly. In the CT scan, a significant decrease in the Hounsfield units and total lung volume was found by echocardiography, and in comparing the two groups, a significant difference in the parameters was noticed. The histopathology demonstrated that the BM-MSCs were able to reduce the infiltration of inflammatory cells and pulmonary hemorrhage and edema.

**Conclusions:**

This study indicated that BM-MSCs play a significant role in the repair of lung injury.

**Electronic supplementary material:**

The online version of this article (10.1186/s13054-018-2272-x) contains supplementary material, which is available to authorized users.

## Background

Lung disorders have significant morbidity and mortality rates worldwide, both in humans and in animals. Acute respiratory distress syndrome (ARDS) is one of the leading causes of respiratory failure around the world. Although early diagnosis, timely medical care, and treatment may lead to improvement of symptoms, the signs return after a period of time. Causes of ARDS are different. It can be caused by direct or indirect damage to the lung epithelium. ARDS is described by severe hypoxemia, decreased pulmonary compliance, diffuse alveolar damage, and bilateral pulmonary infiltrates after cardiac edema [1, 2] and confirmed by a combination of clinical, physiological, and chest imaging parameters. Pulmonary inflammation with disruption of the mechanism of the alveolar–capillary barrier is an important direct cause of ARDS [3, 4]. Therapeutic approaches include mechanical ventilation, neuromuscular blocking agents, fluid management, drug and antimicrobial therapy, and prone positioning [3, 5, 6]. These therapeutic strategies have a supportive role and cannot prevent the progression of the disease [7–10]. The ultimate approach is lung transplant, but it has many problems for recipient patients such as lack of suitable donors and the use of immunosuppressive drugs over a lifetime to prevent rejection of the transplant [11]. Therefore, the recognition of new therapeutic approaches such as stromal cell therapy is essential [12]. MSCs confer immunomodulatory and anti-inflammatory effects, enhance bacterial clearance, reduce cell injury and death, and are angiogenic [5, 13]. The mechanism of the MSCs’ effects includes several pathways mediated through differentiation, proliferation, soluble intermediate release, extracellular vesicles, transfer of organelles, and direct cell-to-cell contact, which decrease activation of inflammatory cell secretion of paracrine mediators [14, 15]. Recent studies have shown positive effects of MSC-based therapy for ARDS. Induction of inflammation by the LPS of *Escherichia coli* O55:B5 is one of the best and simplest methods for making an experimental model of ARDS. Although the ARDS animal models cannot reflect human ARDS accurately, the rabbit model is similar and hence suitable for translating the results from pilot to clinical conditions [16]. Anatomical, physiological, genetic, and biochemical similarity to humans simulates human lung disease, and as the rabbit is easy to handle, it is considered as a suitable model for pulmonary research [17, 18]. Moreover, the rabbit serves as an excellent platform for treatment based on stromal cells [19, 20]. Thus, in this study, the rabbit was used as a model for causing ARDS, and then it was treated with stromal cells. The aim of this study was evaluation of therapeutic potential intrapulmonary administration of BM-MSCs in an experimental model of *E. coli* LPS-induced ARDS in the rabbit.

## Methods

### Isolation, primary culture, and expansion of BM-MSCs

Bone marrow (BM) samples were obtained from the humerus of rabbits in aseptic surgical conditions. After 30 min of centrifugation (400 relative centrifugal force), mononuclear cells were collected from the interphase, and eventually the cell pellets were seeded into 25-cm^2^ flasks (SPL Life Sciences, Pocheon, South Korea) with DMEM-high glucose, 20% FBS (Life Technologies, Carlsbad, CA, USA), and 100 U/ml penicillin/streptomycin (Biowest, Nuaillé, France) and incubated at 37 °C in humid air with 5% CO_2_ (Memmert, Eagle, WI, USA). When the adhesion of the cells was near confluence (more than 70%), the cells were trypsinized by trypsin-ethylenediaminetetraacetic acid of 0.25% (Life Technologies) and replated at dilutions of 1:2 under conditions of the same cultivation. The characteristics of the BM-MSCs were labeled with phycoerythrin-conjugated antibodies against CD45 (BioLegend, San Diego, CA, USA), CD90 (eBioscience, San Diego, CA, USA) and CD34 and CD29 (Abcam, Cambridge, UK), and the multilineage differentiation ability of BM-MSCs to engage in osteogenic and adipogenic differentiation was checked in vitro. This is described in more detail in the additional files.

### Experimental design

#### ARDS experimental model

Ten healthy adult male New Zealand white rabbits were chosen, and an ARDS experimental model was induced with LPS from *E. coli* O55:B5 [21] (Sigma-Aldrich, St. Louis, MO, USA) at 400 μg/kg dissolved in 0.1 ml of PBS intrapulmonary the under bronchoscopic guidance. After the ARDS confirmation, rabbits were randomly distributed into two groups: (1) the control group (ARDS + PBS) and (2) the treatment group (ARDS + BM-MSC). Protocol details are available in the additional files.

#### BM-MSC autologous transplant

A total of 10^10^ BM-MSCs suspended in 0.1 ml of PBS [5] were autologously transplanted intrapulmonary under bronchoscopic guidance 24 h after induction of ARDS. Details of the method are provided in the additional files.

### Analyses

#### Clinical assessment

During the study, the clinical signs of rabbits were calculated and recorded on the basis of clinical scores for each rabbit. Heart rate (HR), respiratory rate (RR), body temperature, twitch, abnormal breathing, nasal discharge, cough, appetite, and physical condition were measured using a clinical score. The scoring is based on clinically evaluated criteria that were individually defined and measured for each rabbit (Additional file 1: Table S1).

### Imaging

#### Computed tomography and echocardiography

Computed tomographic (CT) scans of the lung of rabbits were taken with the SOMATOM Spirit Class II (Siemens, Erlangen, Germany), and echocardiographic examinations were performed using a Vivid 7 ultrasound system (GE Healthcare, Milwaukee, WI, USA) with a 4.4–10.0-MHz phased-array transducer (10S) during experimental modeling of ARDS before and 12, 24, 48, 72, and 168 h after transplant in each animal under the same circumstances. More details are provided in the additional files.

### Sampling

#### Blood and bronchoalveolar lavage samples

Blood samples were collected from the central ear artery for blood gas analyses using blood gas analyzers (OPTI CCA-TS; OPTI Medical Systems, Roswell, GA, USA) and from the ear vein for hematologic parameter analysis and measurement of cytokines (tumor necrosis factor [TNF]-α, interleukin [IL]-6, and IL-10) with a commercially available enzyme-linked immunosorbent assay kit (EASTBIOPHARM, Hangzhou, China) before transplant of BM-MSCs and then for 3, 6, 12, 24, 48, 72, and 168 h after transplant. Also, bronchoalveolar lavage (BAL) samples were collected by fiberoptic bronchoscope (11262 BC; Karl Storz, Tuttlingen, Germany) before and 24, 48, 72, and 168 h after transplant. Then, the centrifuged BALs were stored at − 80 °C for measurement of cytokines. Protocol details are available in the additional files.

### Histopathology

The rabbits were killed 7 days after BM-MSC transplant. First, the lungs and hearts were macroscopically examined, and then sections of them were routinely prepared, stained with H&E, and observed by use of an E600 Eclipse optical microscope (Nikon Instrument, Tokyo, Japan). More details are provided in the additional files.

### Statistical analysis

The results were analyzed statistically using IBM SPSS Statistics version 24 software (IBM, Armonk, NY, USA). For variables in this study, data were analyzed with the repeated measures independent samples *t* test, Friedman test, and Mann-Whitney *U* test, and *p* < 0.05 was considered statistically significant.

## Results

### Characterization of BMSCs

#### Culture of BM-MSCs

The plastic adherent BM-MSCs proliferated 5–7 days after seeding and reached 80% confluence about 2 weeks later. After three passages, the adherent cells were observed by microscopy to display homogeneous spindle fibroblast-like morphology (Additional file 1: Figure S1).

#### Flow cytometric analysis

Flow cytometric analysis demonstrated that cultured BM-MSCs expressed a particular pattern of cell surface markers of CD29 and CD90, 92% and 89%, respectively, but were uniformly negative for CD34 and CD45 (Additional file 1: Figure S2), which indicates cultured adherent cells were MSCs with high purity. Thus, the pure MSCs whose immunophenotype was confirmed were used in this study.

#### Differentiation

Multilineage differentiation ability of BM-MSCs to engage in osteogenic and adipogenic differentiation in vitro confirmed potential pluripotent MSCs, and their ability to form osteoblasts and adipocytes when incubated in differentiation medium was retained (Additional file 1: Figure S3).

### Confirmation of ARDS experimental model

Twenty-four hours after the intrapulmonary administration of LPS, inflammation and edema were stabilized in the lung. Two different evaluations proved ARDS occurrence: (1) Clinical examination showed changes in respiratory sounds during auscultation, such as crackle and wheeze, increased respiratory rate/hyperpnea (*p* = 0.004), heart rate/tachycardia (*p* = 0.008) and body temperature/hyperthermia (*p* = 0.011), cough, mucus hyperemia, abnormal discharge, and reduced appetite; and (2) plain chest radiograph showing significant bilateral radiologic density (air bronchogram and air alveologram patterns and lung edema and bronchial and bronchiolar septum thickness) were also confirmed (Additional file 1: Figure S4). These results confirmed the experimental model of ARDS compared with the baseline 24 h after injection of LPS.

### Clinical and paraclinical findings after transplant of BM-MSCs in an experimental model of ARDS

#### Improved clinical signs with MSCs

**Fig. 1:**
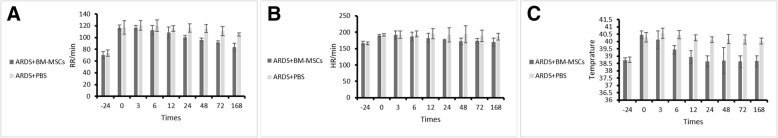
Vital signs of rabbits (mean ± SD) in the treatment (acute respiratory distress syndrome [ARDS] + bone marrow mesenchymal stem cells) and control (ARDS + PBS) groups at the different time points. **a** Respiratory rate. **b** Heart rate. **c** Body temperature

According to the statistical analysis, reduction of RR in the treatment group was significant at 24 h (*p* = 0.002), 48 h (*p* = 0.036), 72 h (*p* = 0.037), and 168 h (*p* = 0.042) after BM-MSC transplant compared with time 0 (before BM-MSC transplant). HR reduction was significant at 24 h (*p* = 0.047) after transplant compared with time 0, but in RR and HR in the control group, it was not significantly different at various times. The RR results of the comparison between two groups showed a significant difference at 24 h (*p* = 0.028), 48 h (*p* = 0.03), 72 h (*p* = 0.01), and 168 h (*p* = 0.044) (Fig. 1 and Additional file 1: Table S2). The body temperature change in rabbits of the treatment group was significant at 6 h (*p* = 0.010), 12 h (*p* = 0.016), 24 h (*p* = 0.044), 72 h (*p* = 0.044), and 168 h(*p* = 0.043) after transplant compared with time 0. Also, the comparison between two groups showed significant differences at 12 h (*p* = 0.047), 24 h (*p* = 0.021), 48 h (*p* = 0.035), and 168 h (*p* = 0.037) (Fig. 1 and Additional file 1: Table S2).

Changes in respiratory sounds (including crackle, wheeze, friction sounds) in the treatment group were significant compared with the control group at 24 h (*p* = 0.50), 48 h (*p* = 0.014), 72 h (*p* = 0.017), and 168 h (*p* = 0.014) after transplant. Comparison of appetite between the two groups displayed a positive and significant association at 12 h (*p* = 0.002), 24 h (*p* = 0.014), 48 h (*p* = 0.007), 72 h (*p* = 0.004), and 168 h (*p* = 0.004) after transplant.

In both groups, after inflammation, unilateral or bilateral mucosal secretions from the nose were produced that were occasionally accompanied by color changes. But after transplant of BM-MSCs, statistical comparison showed a significant decrease in nasal discharge compared with the control group at 24 h (*p* = 0.011), 48 h (*p* = 0.007), 72 h (*p* = 0.007), and 168 h (*p* = 0.008). The regular rhythm of nasal twitching in the rabbit is a reason for the rabbit’s health and alertness. Nasal twitching was reduced at the time of inflammation. But there was a significant difference between results at 12 h (*p* = 0.005), 24 h (*p* = 0.005), 72 h (*p* = 0.014), and 168 h (*p* = 0.014) after transplant.

Also, there was a significant decrease in cough count between the two groups at 12 h (*p* = 0.014), 24 h (*p* = 0.005), 48 h (*p* = 0.006), 72 h (*p* = 0.004), and 168 h (*p* = 0.005). Regarding the statistical analysis, comparison of mucous membranes (conjunctiva, palpebra tertia, gingiva, rectum) in both groups showed hyperemia reduced at 24 h (*p* = 0.031), 48 h (*p* = 0.011), 72 h (*p* = 0.005), and 168 h (*p* = 0.005) after BM-MSC transplant. After inflammation, rabbits showed depression and delay in response to environmental stimuli. But transplant of BM-MSCs indicated a significant improvement in consciousness at 24 h (*p* = 0.017), 48 h (*p* = 0.017), 72 h (*p* = 0.004), and 168 h (*p* = 0.005).

### MSCs cause blood cells and BAL cells to balance

#### Blood cells

The measured blood parameters at different times are shown in the Table 1. Rabbits in the two groups had significant leukocytosis 1 day after inflammation (*p* < 0.005). But the BM-MSC transplant reduced the number of white blood cells. The changes were significant at 12 h (*p* = 0.046), 24 h (*p* = 0.019), 48 h (*p* = 0.022), 72 h (*p* = 0.044), and 168 h (*p* = 0.043) compared with time 0. Also, comparison of the two groups showed that cell transplant was effective at 6 h (*p* = 0.043), 12 h (*p* = 0.000), and 24 h (*p* = 0.047) (Table 1).

**Table 1:** Hematological parameters

Hematological parameters	Group	− 24 (h)	0 h	3 h	6 h	12 h	24 h	48 h	72 h	168 h
WBC (/μl)	Treatment	*8240 ± 1469.01	13,760 ± 890.50	*13,040 ± 1228.00	#*12,340 ± 1377.67	#10,740 ± 89.44	#9940 ± 54.77	9580 ± 396.23	9240 ± 1163.18	8700 ± 1437.01
	Control	*7720 ± 810.55	13,160 ± 522.49	*13,020 ± 756.30	*12,520 ± 589.06	*12,720 ± 1151.95	*12,620 ± 630.07	*12,640 ± 702.13	*12,380 ± 690.65	*11,740 ± 1040.67
SEG (/μl)	Treatment	*2582 ± 937.64	8355.20 ± 885.64	#*7468.80 ± 1351.25	#*6441.60 ± 279.25	#5814 ± 541	#5098 ± 84.81	#3828.20 ± 246.90	3653.80 ± 205.11	3156.40 ± 1657.72
	Control	*2550.20 ± 843.41	8467.60 ± 992.59	*8512.20 ± 599.36	*8101.20 ± 954.74	*7826.60 ± 1288.89	*7739.80 ± 1000.22	*7654.20 ± 1395.41	*7299.40 ± 533.47	*6676.20 ± 450.88
BAND (/μl)	Treatment	27.4 ± 26.43	73 ± 56.74	71.6 ± 66.72	64 ± 58.99	60 ± 56.56	58.8 ± 38	#55.2 ± 40	#44 ± 36.29	#35 ± 22.91
	Control	34.8 ± 32.42	73.2 ± 57.99	77.6 ± 49.88	86.2 ± 52.50	89.2 ± 57.47	102 ± 63.80	112.8 ± 81.68	131.2 ± 104.88	*174.8 ± 51.89
LYM (/μl)	Treatment	5173.4 ± 577.66	3363.4 ± 900.92	3480.4 ± 1197.64	3758 ± 664.16	3885.8 ± 606.60	4120.6 ± 743.79	4255.2 ± 891.68	4509 ± 994.02	4906.8 ± 1015.92
	Control	4852.4 ± 1214.24	3175.6 ± 882.11	3268 ± 1603.72	3376 ± 813.13	3393.6 ± 1145.60	3471.8 ± 1196.61	3596.2 ± 1301.70	3678.4 ± 1338.22	3795.8 ± 815.30
MON (/μl)	Treatment	86 ± 5.47	76 ± 60.76	80 ± 109.54	78 ± 174.41	76 ± 66.55	80 ± 123.28	78 ± 126.17	78 ± 62.60	#80 ± 77.78
	Control	76 ± 4.18	68 ± 8.36	72 ± 97.31	78 ± 83.18	84 ± 85.02	90 ± 108.39	96 ± 108.30	100 ± 70.71	*130 ± 12.24
RBC (×10^6^/μl)	Treatment	6.11 ± 0.21	6.23 ± 0.90	6.28 ± 0.78	6.28 ± 0.67	6.24 ± 1	6.2 ± 0.92	#6.18 ± 0.96	6.13 ± 0.98	6.08 ± 0.99
	Control	6.07 ± 0.09	6.21 ± 1.11	6.27 ± 0.91	6.29 ± 1.09	6.32 ± 0.88	6.32 ± 0.86	*6.35 ± 0.09	6.31 ± 1.20	6.24 ± 1.04
Hgb (g/dl)	Treatment	12.12 ± 0.44	12.44 ± 0.56	12.56 ± 1.56	12.48 ± 1.16	12.36 ± 0.51	12.34 ± 1.32	#12.26 ± 0.94	12.2 ± 1.01	12 ± 0.57
	Control	12 ± 0.18	12.22 ± 1.46	12.52 ± 1.86	12.72 ± 1.97	12.88 ± 1.6	12.9 ± 1.50	*13.04 ± 0.11	12.86 ± 1.10	12.62 ± 0.52
Hct (%)	Treatment	39.16 ± 2.24	40.66 ± 2.66	40.58 ± 3.20	40.46 ± 2.36	40.18 ± 2.43	40.1 ± 2.87	39.54 ± 2.27	39.3 ± 2.74	39.46 ± 2.08
	Control	38.9 ± 2.54	40.4 ± 2.67	40.68 ± 3.29	40.9 ± 4.04	40.94 ± 4.18	41.02 ± 3.54	40.7 ± 3.45	40.52 ± 3.07	40.2 ± 3.29
Plt (×10^3^/μl)	Treatment	371.2 ± 87.08	353 ± 74.24	339 ± 80.56	347.6 ± 64.43	345.6 ± 58.65	361.8 ± 74.88	380.4 ± 75.92	374 ± 66.60	368.4 ± 61.05
	Control	339 ± 103.84	330.2 ± 111.21	322.4 ± 78.15	322 ± 104.41	324 ± 42.95	316.8 ± 70.25	337.2 ± 64.13	322.8 ± 74.38	332.4 ± 82.26

*Abbreviations: WBC* White blood cells, *SEG* Segmented heterophils, *BAND* Band heterophils, *LYM* Lymphocytes, *MON* Monocytes, *RBC* Red blood cells, *Hgb* Hemoglobin, *Hct* Hematocrit, *Plt* Platelets

Data are presented as mean ± SD for rabbits in the treatment (acute respiratory distress syndrome [ARDS] + bone marrow mesenchymal stem cells) and control (ARDS + PBS) groups at the different time points of sampling

*p ≤ 0.05; significant compared with inflammation time in the same group

#*p* ≤ 0.05; significant compared with the control group at the same time

The transplant of BM-MSCs reduced the heterophil count following ARDS. Changes were significant at 12 h (*p* = 0.048), 24 h (*p* = 0.049), 48 h (*p* = 0.009), 72 h (*p* = 0.007), and 168 h (*p* = 0.024) against inflammation time (time 0). The comparison between two groups showed significant differences at 3 h (*p* = 0.020), 6 h (*p* = 0.042), 12 h (*p* = 0.030), 24 h (*p* = 0.001), and 48 h (*p* = 0.046) (Additional file 1: Figure S5).

Comparison of heterophil band numbers between the two groups showed that cell transplant was affected at 48 h (*p* = 0.046), 72 h (*p* = 0.021), and 168 h (*p* = 0.038).

Statistical analysis showed that there was no significant difference in the lymphocyte count, monocytes, platelets, hematocrit in or between the treatment and control groups during the study. There was a significant difference in the number of red blood cells (*p* = 0.007) and concentration of hemoglobin (*p* = 0.027) in the treatment group compared with the control group only at 48 h.

#### Cells from BAL

**Fig. 2:**
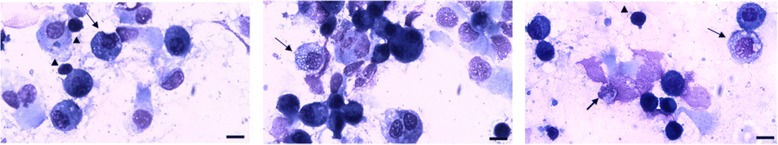
Determination of complete cell counts in bronchoalveolar lavage (BAL) samples of the rabbit. Using Wright-Giemsa staining, we counted inflammatory cells in the BAL samples of the control group among the total of 100 cells. Macrophages (*arrow*), heterophils (*thick arrow*), lymphocyte (*arrowhead*). Bars = 50 μm

In BAL, the total nucleated cell count included ciliated epithelial cells, squamous epithelial cells, alveolar macrophages, leukocytes, heterophils, eosinophils, and plasma cells. Typically, total leukocyte cells in treatment group included macrophages and lymphocytes, and in the control group, they included macrophages and heterophils (Fig. 2).

**Fig. 3:**
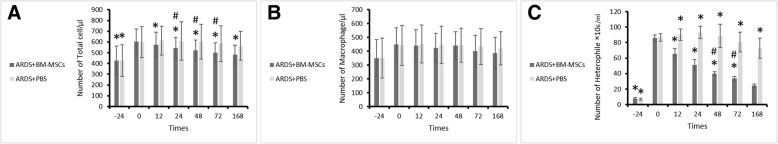
The number of bronchoalveolar lavage cells of rabbits (mean ± SD) in the treatment (acute respiratory distress syndrome [ARDS] + bone marrow mesenchymal stem cells) and control (ARDS + PBS) groups during the different times of sampling. **a** Total cells. **b** Macrophages. **c** Heterophils

The results demonstrated that the BAL cell count was significantly increased in inflammatory conditions, but transplant of stromal cells caused the inflammatory cells to balance so that, in the treatment group, decrease of the total cell count was significant at all times except time 0 (*p* < 0.05), and there was a significant reduction in the total cell count compared with the control group at 24 h (*p* = 0.045), 48 h (*p* = 0.46), and 72 h (*p* = 0.048). Also, BM-MSCs were able to significantly reduce the number of alveolar heterotrophs, as well as the number of macrophages in BAL, so that decrease of heterotrophs was significant at 12 h (*p* = 0.03), 24 h (*p* = 0.01), 48 h, 72 h, and 168 h (*p* = 0.000) against inflammation time and at 48 h (*p* = 0.049), 72 h (*p* = 0.031), and 168 h (*p* = 0.042) compared with the control group. Also, reduction in macrophages was significant at 72 h (*p* = 0.033) and 168 h (*p* = 0.005) compared with time 0. No significant change in the number of lymphocytes was observed in or between the two groups (Fig. 3) (Additional file 1: Table S3).

### Regulation of arterial blood gases with MSCs

The partial pressure of oxygen (PO_2_) and O_2_ saturation (SatO_2_) levels were decreased, and partial pressure of carbon dioxide (PCO_2_) levels were increased, in ARDS (time 0) compared with baseline (− 24 h), significantly. Following BM-MSC transplant, PO_2_ increment was significant at 12 h (*p* = 0.043), 24 h (*p* = 0.005), and 48 h (*p* = 0.005), and comparison between the two groups demonstrated that severe hypoxemia was significantly recovered after transplant at 12 h (*p* = 0.027), 24 h (*p* = 0.042), and 48 h (*p* = 0.040).

Also, following transplant of BM-MSCs, changes of SatO_2_ were significant at 12 h (*p* = 0.038), 24 h (*p* = 0.030), 48 h (*p* = 0.009), 72 h (*p* = 0.007), and 168 h (*p* = 0.024) compared with time 0, and comparison of the two groups showed that cell transplant was effective at 3 h (*p* = 0.020), 6 h (*p* = 0.042), 12 h (*p* = 0.030), 24 h (*p* = 0.001), and 48 h (*p* = 0.046) (Additional file 1: Table S4).

Additionally, PCO_2_ was significantly decreased in the treatment group at 24 h (*p* = 0.036), 48 h (*p* = 0.034), and 72 h (*p* = 0.01) after transplant, and comparison between the two groups displayed a significant decrease of PCO_2_ at 48 h and 72 h (*p* = 0.016).

**Fig. 4:**
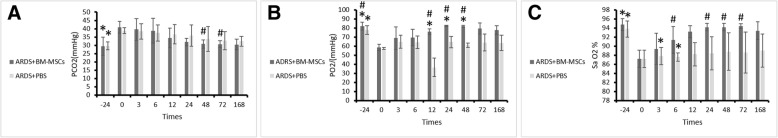
Arterial blood gas analysis of rabbits (mean ± SD) in the treatment (acute respiratory distress syndrome [ARDS] + bone marrow mesenchymal stem cells) and control (ARDS + PBS) groups during the different times of sampling. **a** Partial pressure of carbon dioxide. **b** Partial pressure of oxygen. **c** O_2_ saturation

Also, statistical analysis for pH value indicated significant differences between the two groups at 24 h (*p* = 0.019). Respiratory acidosis occurred in both groups at 3 and 6 h via pH decrease and increase in PCO_2_. Analysis of bicarbonate, base excess, and anion gap data showed no significant difference in and between the two groups (Fig. 4).

### Effect of MSCs on arterial blood electrolytes

The results showed nonsignificant reduction in the value of Na^+^, K^+^, and Cl^−^ in and between the treatment and control groups at the different times and alone. There was a significant difference in the Cl^−^ value in the treatment group compared with the control group at 24 h (*p* = 0.029) (Additional file 1: Table S5).

### Reduced levels of proinflammatory cytokines and increase in anti-inflammatory cytokine by MSCs

Duplicated measurements of each variable were performed, and the average of the data was obtained. The measured cytokine levels in plasma and BAL samples at different times are shown in Additional file 1: Table S6. BM-MSC transplant decreased proinflammatory cytokines (TNF-α and IL-6) and increased anticytokine inflammation (IL-10) during endotoxin injury in plasma and BAL.

After transplant of MSCs, the concentration of the TNF-α in BAL decreased at 12 h (*p* = 0.035), 24 h (*p* = 0.011), 48 h (*p* = 0.007), and 168 h (*p* = 0.014) compared with time 0, significantly, and comparison of the two groups showed that cell transplant was affected at times of 12 h (*p* = 0.031), 24 h (*p* = 0.018), 48 h (*p* = 0.009), and 168 h (*p* = 0.014). Also, the levels of TNF-α in plasma were recovered with MSCs at 24 h (*p* = 0.040), 48 h (*p* = 0.006), 72 h (*p* = 0.002), and 168 h (*p* = 0.003) against inflammation time (0 h), significantly, and comparison of the two groups showed significant difference at 72 h (*p* = 0.008) and 168 h (*p* = 0.025).

The IL-6 concentration was downregulated by MSCs, so that the levels of IL-6 in the BAL were significantly lower at times of 12 h (*p* = 0.047), 24 h (*p* = 0.011), 48 h (*p* = 0.001), and 168 h (*p* = 0.041) than time 0. Comparison of the two groups indicated significant differences at 12 h (*p* = 0.032), 24 h (*p* = 0.018), 72 h (*p* = 0.041), and 168 h (*p* = 0.008). Also, the concentration of IL-6 was significant in plasma 48 h (*p* = 0.048) and 168 h (*p* = 0.001) after MSC transplant against the inflammation time (0 h), and comparison of the two groups showed that cell therapy was affected at 24 h (*p* = 0.001), 48 h (*p* = 0.005), and 168 h (*p* = 0.012). These results demonstrated the immunomodulatory potential of these BM-MSCs.

**Fig. 5:**
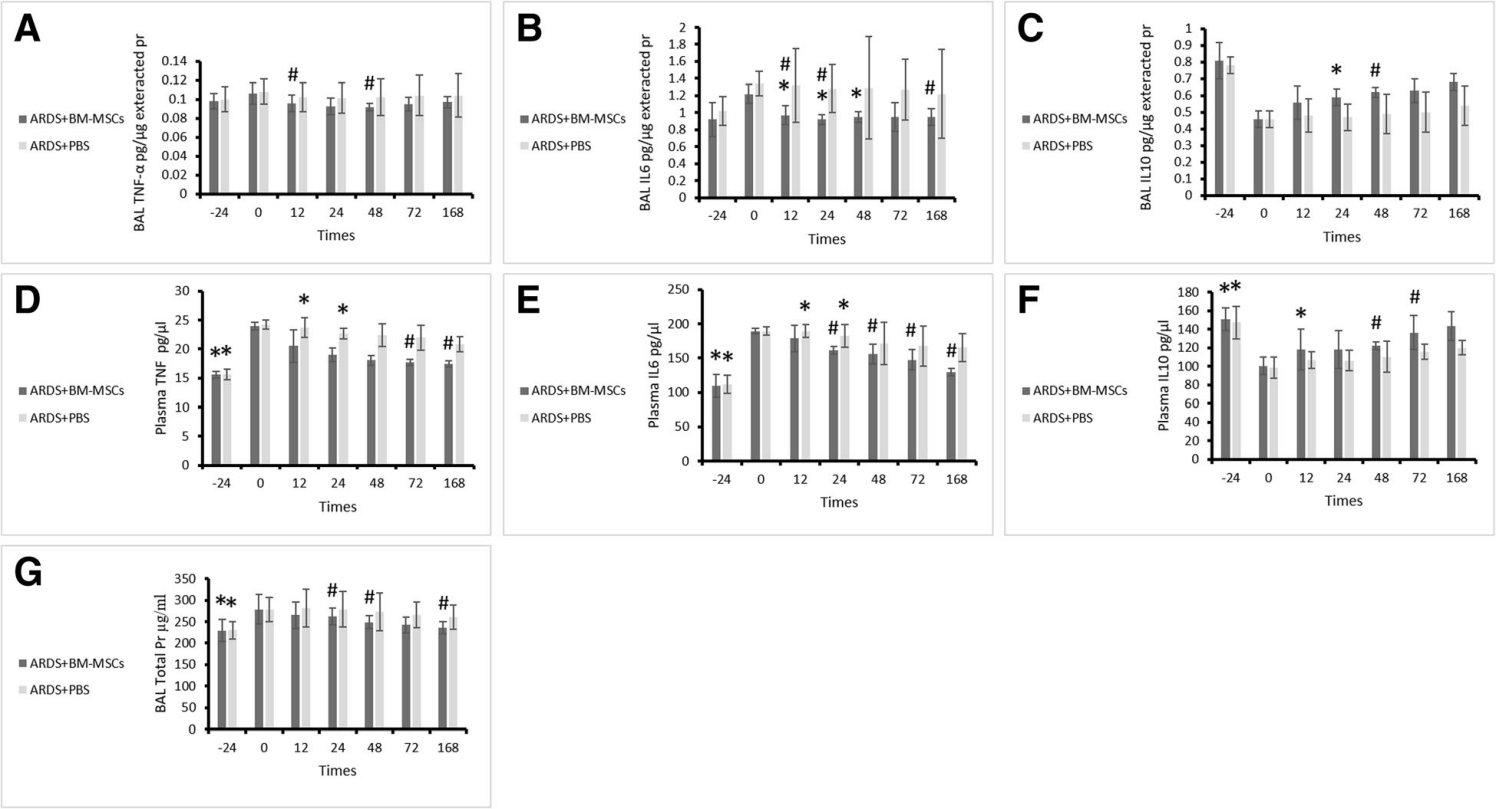
Amount of cytokines in bronchoalveolar lavage and plasma of rabbits (mean ± SD) in the treatment (acute respiratory distress syndrome [ARDS] + bone marrow mesenchymal stem cells) and control (ARDS + PBS) groups at the different times of sampling. **a**–**c** BAL samples: **a** Tumor necrosis factor (TNF)-α. **b** Interleukin (IL)-6. **c** IL-10. **d**–**f** Plasma samples: **d** TNF-α. **e** IL-6. **f** IL-10

In contrast, when MSCs were administered, IL-10 was significantly increased in the BAL and plasma. Concentration of IL-10 in BAL was significant at 12 h (*p* = 0.047), 24 h (*p* = 0.011), 48 h (*p* = 0.001), and 168 h (*p* = 0.041) compared with time 0, and comparison of the two groups showed significant differences at 12 h (*p* = 0.032), 24 h (*p* = 0.018), 72 h (*p* = 0.041), and 168 h (*p* = 0.008) (Fig. 5). Also, increase of IL-10 concentration in plasma was significant at 48 h (*p* = 0.047), 72 h (*p* = 0.044), and 168 h (*p* = 0.022) against time 0. MSCs also increased plasma IL-10 concentrations compared with control group at 48 h (*p* = 0.043) and 72 h (*p* = 0.029), significantly (Fig. 5), so that BMSCs reduced lung injury and inflammation via significant immunomodulatory properties and attenuated the severity of ARDS.

### Imaging findings

#### Tomodensitometric and volumetric findings of lung CT scans

**Fig. 6:**
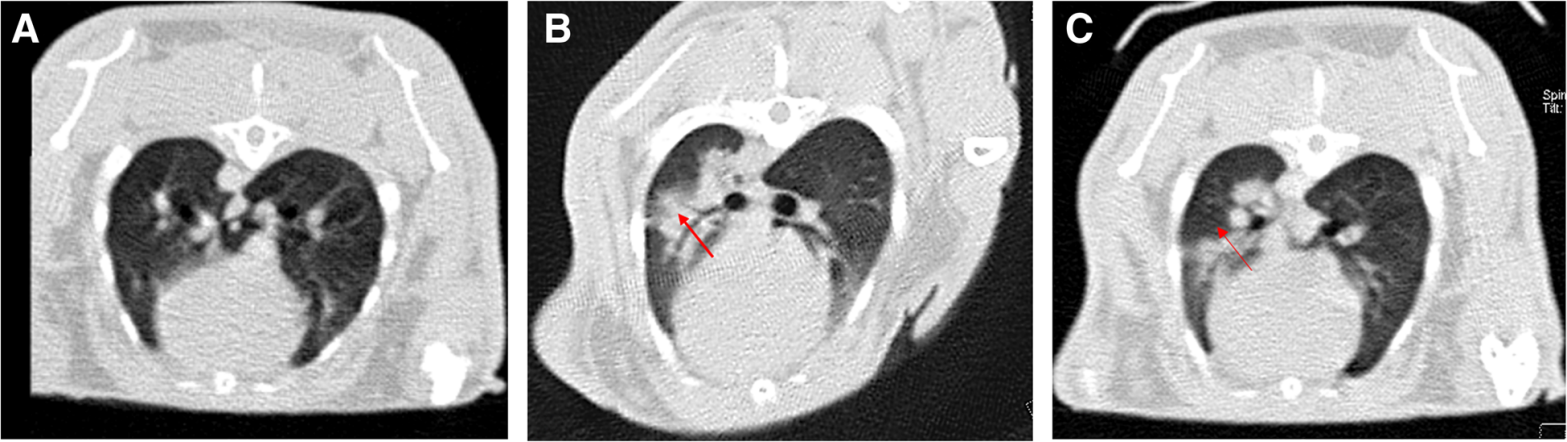
High-resolution computed tomographic scans of the thorax (lung window) in the rabbit. **a** Transverse section of lung. **b** Increased attenuation due to inflammation in the lung of the control group. Notice the typical air bronchogram (alveolar lung pattern). **c** Decreased attenuation in the lung of the treatment group (recipient of bone marrow mesenchymal stem cells). The dorsal regions of the lung have the highest volume

**Fig. 7:**
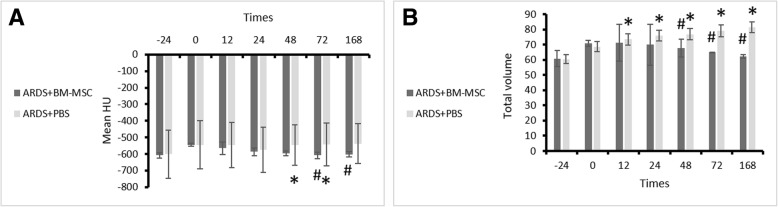
The amount of computed tomographic scan volumetry (mean Hounsfield units and total volume) of rabbits (mean ± SD) in the treatment (acute respiratory distress syndrome [ARDS] + bone marrow mesenchymal stem cells) and control (ARDS+PBS) groups at the different time points. **a** Mean Hounsfield units. **b** Total volume

Hounsfield units and volumes of the aerated and nonaerated areas of the right and left lungs were measured on CT scans. Quantitative estimation (the Hounsfield unit measurement) was done for different adjacent CT sections with the Leonardo workstation and software tools (Siemens). Lung parenchymal margins were manually demarcated, and then average Hounsfield units were obtained for each section. Also, the 3D pattern was observed for a better evaluation of the lung parenchyma (data not shown). These measurements demonstrated that Hounsfield units had increased 1 day after the experimental inflammation (before stromal cells therapy), which represents replacement of alveolar air with mucous and inflammatory cells (Fig. 6). A significant decrease in the Hounsfield units was seen at 48 h (*p* = 0.032), 72 h (*p* = 0.036), and 168 h (*p* = 0.025) post-transplant, which indicates an increase in aerated volume of the lung in the treatment group. Also, variation volumes were compared and showed that total lung volume (aerated + nonaerated + tissue + edematous fluids) in both groups increased after ARDS, but transplant of BM-MSCs had decreased the process at 72 h (*p* = 0.047) and 168 h (*p* = 0.027). On CT scans, most nonaerated areas were observed in lower lobes in the caudoventral area (Fig. 7 and Additional file 1: Table S7).

### Echocardiography findings

The images and amounts of Doppler and M-mode echocardiographic parameters are shown in Additional file 1: Figure S6. The amounts of BAL and plasma cytokines of rabbits are shown in Fig. 6 and Table 2, respectively. Echocardiographic data showed no significant difference in the treatment group (BM-MSC recipients), but comparison of the two groups revealed that percentage ejection fraction at 72 h (*p* = 0.042) and 168 h (*p* = 0.038), percentage fractional shortening at 72 h (*p* = 0.049) and 168 h (*p* = 0.044), interventricular septal end diastole at 48 h (*p* = 0.014), 72 h (*p* = 0.038), and 168 h (*p* = 0.042), interventricular septal end systole at 24 h (*p* = 0.005), 48 h (*p* = 0.009), and 72 h (*p* = 0.010), left ventricular internal dimension systole at 72 h (*p* = 0.048) and 168 h (*p* = 0.022), aortic root diameter at 72 h (*p* = 0.027) and 168 h (*p* = 0.042), left atrial diameter at 168 h (*p* = 0.048), left ventricular outflow tract maximum velocity at 168 h (*p* = 0.041) and right ventricular outflow tract maximum velocity at 168 h (*p* = 0.039) had significant differences.

**Table 2:** M-mode and Doppler echocardiography parameters

Time (h)	Group	− 24 h	0 h	12 h	24 h	48 h	72 h	168 h
Parameters								
EDV (mm)	Treatment	2.5 ± 0.82	3.01 ± 0.86	3.76 ± 1.68	4 ± 1.09	3.87 ± 0.88	3.42 ± 0.97	3.24 ± 0.29
	Control	2.65 ± 1.11	3.49 ± 0.72	3.92 ± 1.52	3.38 ± 0.79	2.95 ± 0.67	3.02 ± 0.75	3.13 ± 0.73
EF (%)	Treatment	57.6 ± 9.3	63.1 ± 6.6	68.96 ± 15.5	60.95 ± 9.5	57.45 ± 12.92	63.03 ± 7.72	61.38 ± 3.7
	Control	56.33 ± 6.6	62.99 ± 5.6	65.02 ± 14.86	67.52 ± 17.68	59.88 ± 12.24	#55.03 ± 20.01	#51.1 ± 18.4
ESV (mm)	Treatment	0.89 ± 0.25	1.13 ± 0.53	1.22 ± 1.24	1.09 ± 0.74	1 ± 0.25	1.06 ± 0.46	1.05 ± 0.49
	Control	0.93 ± 0.79	1.25 ± 0.46	1.3 ± 0.27	1.15 ± 0.69	1.06 ± 0.49	1.04 ± 0.4	1.02 ± 0.3
FS (%)	Treatment	33.72 ± 10.58	36.92 ± 8.26	35.01 ± 12.47	37.09 ± 13.47	35.24 ± 7.24	34.81 ± 6.12	34.91 ± 4.11
	Control	35.1 ± 12.74	38.58 ± 9.5	37.5 ± 5.78	36 ± 3.39	33.8 ± 10.41	#31.01 ± 15.03	#28.41 ± 16.22
IVSd (mm)	Treatment	0.25 ± 0.06	0.27 ± 0.047	0.27 ± 0.027	0.26 ± 0.065	0.25 ± 0.037	0.26 ± 0.045	0.25 ± 0.08
	Control	0.37 ± 0.13	0.38 ± 0.083	0.37 ± 0.073	0.41 ± 0.1	#0.34 ± 0.17	#0.35 ± 0.12	#0.35 ± 0.22
IVSs (mm)	Treatment	0.37 ± 0.19	0.39 ± 0.07	0.4 ± 0.04	0.35 ± 0.04	0.36 ± 0.06	0.34 ± 0.08	0.33 ± 0.12
	Control	0.51 ± 0.13	0.59 ± 0.18	0.59 ± 0.23	0.66 ± 0.3	#0.57 ± 0.35	#0.55 ± 0.34	#0.57 ± 0.3
LVIDd (mm)	Treatment	0.99 ± 0.09	1.11 ± 0.12	1.18 ± 0.23	1.25 ± 0.11	1.26 ± 0.11	1.29 ± 0.24	1.16 ± 0.02
	Control	1 ± 0.18	1.19 ± 0.09	1.37 ± 0.31	1.19 ± 0.11	1.13 ± 0.09	#1.17 ± 0.14	#1.18 ± 0.12
LVIDs (mm)	Treatment	0.55 ± 0.1	0.77 ± 0.13	0.87 ± 0.36	0.74 ± 0.25	0.64 ± 0.06	0.66 ± 0.04	0.6 ± 0.05
	Control	0.57 ± 0.28	0.88 ± 0.14	0.88 ± 0.28	0.79 ± 0.22	0.76 ± 0.13	0.75 ± 0.18	0.73 ± 0.57
LVPWd	Treatment	0.39 ± 0.06	0.31 ± 0.09	0.29 ± 0.04	0.28 ± 0.05	0.28 ± 0.03	0.31 ± 0.06	0.3 ± 0.08
	Control	0.31 ± 0.1	0.33 ± 0.061	0.29 ± 0.06	0.35 ± 0.08	0.38 ± 0.08	0.36 ± 0.08	0.34 ± 0.08
LVPWs	Treatment	0.48 ± 0.1	0.46 ± 0.09	0.39 ± 0.1	0.47 ± 0.14	0.43 ± 0.14	0.48 ± 0.16	0.41 ± 0.7
	Control	0.42 ± 0.1	0.44 ± 0.16	0.46 ± 0.09	0.46 ± 0.09	0.57 ± 0.1	0.56 ± 0.13	0.54 ± 0.17
SV (ml)	Treatment	1.71 ± 0.41	1.87 ± 0.48	2.1 ± 0.74	2.76 ± 0.36	2.44 ± 0.62	2.36 ± 0.82	2.19 ± 0.79
	Control	2.16 ± 0.61	2.23 ± 0.44	2.61 ± 1.47	2.22 ± 0.22	1.89 ± 0.56	1.98 ± 0.38	2.01 ± 0.6
AO (mm)	Treatment	0.66 ± 0.047	0.6 ± 0.12	0.62 ± 0.09	0.61 ± 0.11	0.57 ± 0.05	0.67 ± 0.03	0.65 ± 0.02
	Control	0.67 ± 0.06	0.61 ± 0.09	0.59 ± 0.13	0.62 ± 0.16	0.59 ± 0.13	#0.6 ± 0.17	#0.58 ± 0.11
LA (mm)	Treatment	0.83 ± 0.1	0.76 ± 0.07	0.81 ± 0.16	0.85 ± 0.1	0.77 ± 0.19	0.81 ± 0.14	0.89 ± 0.07
	Control	0.84 ± 0.17	0.77 ± 0.02	0.76 ± 0.08	0.88 ± 0.02	0.79 ± 0.11	0.79 ± 0.06	#0.73 ± 0.27
LA/AO	Treatment	1.25 ± 0.15	1.32 ± 0.36	1.34 ± 0.35	1.42 ± 0.18	1.36 ± 0.37	1.19 ± 0.2	1.36 ± 0.05
	Control	1.28 ± 0.34	1.28 ± 0.2	1.3 ± 0.24	1.48 ± 0.42	1.41 ± 0.52	1.43 ± 0.6	1.32 ± 0.62
LVOT_Vmax_ (cm/s)	Treatment	0.57 ± 0.1	0.58 ± 0.14	0.5 ± 0.12	0.51 ± 0.07	0.63 ± 0.08	0.58 ± 0.12	0.56 ± 0.02
	Control	0.57 ± 0.13	0.6 ± 0.08	0.62 ± 0.075	0.61 ± 0.09	0.59 ± 0.03	0.56 ± 0.07	#0.51 ± 0.14
RVOT_Vmax_ (cm/s)	Treatment	0.63 ± 0.08	0.66 ± 0.08	0.58 ± 0.07	0.64 ± 0.14	0.62 ± 0.11	0.62 ± 0.1	0.63 ± 0.09
	Control	0.57 ± 0.08	0.63 ± 0.06	0.64 ± 0.14	0.62 ± 0.09	0.61 ± 0.03	0.59 ± 0.08	#0.54 ± 0.02

*Abbreviations: EDV* End-diastolic volume, *EF* Ejection fraction, *ESV* End-systolic volume, *FS* Fractional shortening, *IVSd* Interventricular septal end diastole, *IVSs* Interventricular septal end systole, *LVIDd* Left ventricular internal dimension diastole, *LVIDs* Left ventricular internal dimension systole, *LVPWd* Left ventricular posterior wall end diastole, *LVPWs* Left ventricular posterior wall end systole, *SV* Stroke volume, *AO* Aortic root diameter, *LA* Left atrium diameter, *LVOT*_*Vmax*_ Left ventricular outflow tract maximum velocity, *RVOT*_*Vmax*_ Right ventricular outflow tract maximum velocity

Data of rabbits are presented as mean ± SD in the treatment (acute respiratory distress syndrome [ARDS] + bone marrow mesenchymal stem cells) and control (ARDS + PBS) groups at the different time points of sampling

#*p* < 0.05; significant compared with the treatment group at the same time

### Findings of gross pathology and histopathology

**Fig. 8:**
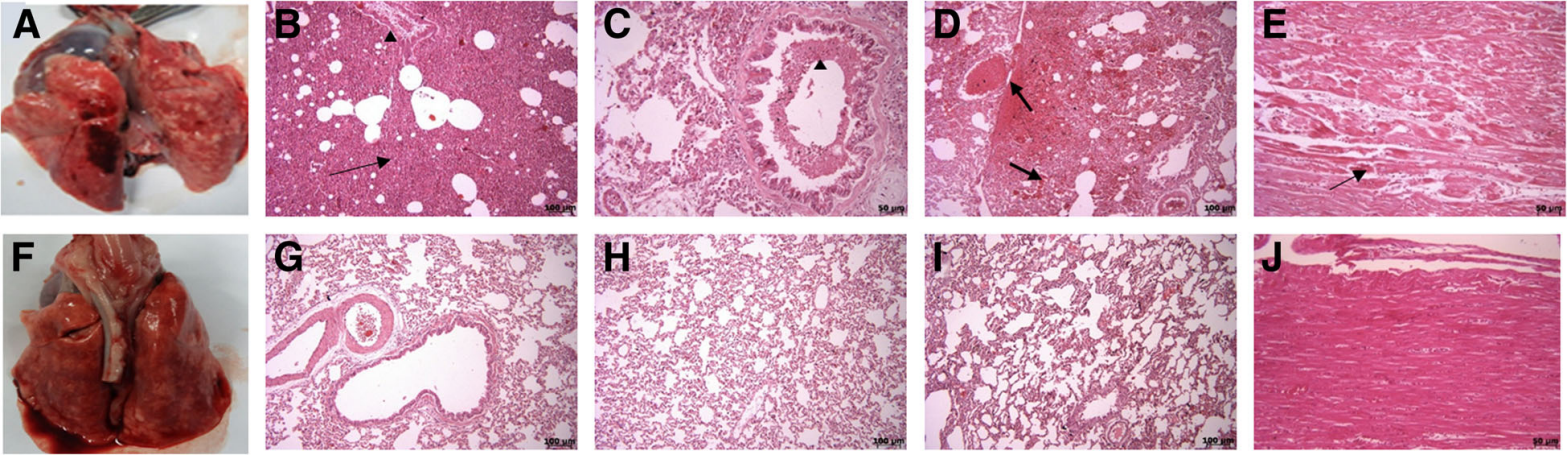
Necropsy and histopathological findings in the rabbit. **a**–**e** Control group. **a** Macroscopic examination of the lung shows hyperemia, hemorrhage, and edema. **b** Interstitial edema and pneumonia (*arrow*). **b** and **c** Inflammatory cell infiltration (*arrowhead*). **d** Hyperemia and severe hemorrhage in alveoli and parenchyma (*arrows*). **e** Myofibrils necrosis of heart (*arrow*). **f**–**k** Treatment group. **f** Macroscopic examination of the lung shows brief hyperemia and edema. **g**–**i** Histopathological examination reveals a decrease in damage in the alveoli and parenchyma of the lung. **k** Lack of damage to the heart. *Note*: In all of the tables, data were presented as mean ± SD (*n* = 5 rabbit per group). *p < 0.05 significant compared with inflammation time in the same group. #*p* < 0.05 significant compared with the control group at the same time

The macroscopic examination of the lungs showed hyperemia, hemorrhage, emphysema, edema, and hepatization in the control group (Fig. 8a), but brief hyperemia and edema were observed in the treatment group (Fig. 8f). Sections of the lung demonstrated different histopathological patterns between the control and treatment groups. Microscopically, lungs showed more severe damage in the control group than the treatment group as hemorrhage in parenchyma and alveoli, moderate to severe vascular hyperemia, moderate to severe interstitial pneumonia, severe alveolar injuries and edema, neutrophilic margination in the capillary vessels, abundant presence of inflammatory cells, epithelial cells and other cell debris (cellularity) in interstitial spaces and alveoli, and thickness of the alveolar septum (Fig. 8b–d). But treatment with BM-MSCs reduced the infiltration rate of inflammatory cells in the alveolar septum, hyperemia, hemorrhage, and edema, and lung structure was approximately normal and only slightly increased the thickness of the alveolar septum. Also, in most sections of the treatment group, injury in the bronchus, bronchioles, and vessels was not observed (Fig. 8g–i).

In macroscopic observations of the heart, no lesions were found in both treatment and control groups, but in the microscopic examination of the heart sections in the control group, necroses were observed in a small number of myofibers (Fig. 8e), whereas in treatment group, no injury was observed (Fig. 8k).

## Discussion

BM-MSCs are an ideal choice for cell therapy because there are fewer complications for cell isolation and also BM autologous cells that are capable of eliminating immune response and transplant rejection [22]. MSCs have positive effects in the repair of ARDS [23].

Although fibroblasts play a role in normal and pathological repair, and an accumulation of fibrocytes, fibroblasts, and myofibroblasts in the alveolar compartment, leading to excessive deposition components of the extracellular matrix but also according to prior findings the effect of them remains controversial [23–25] and unknown, so that reduction of its deposition or enhancement of its degradation could be treatment strategies. [24]

The results of this study showed that the BM-MSCs significantly decreased the severity of clinical symptoms induced by LPS, the number of inflammatory cells in blood and BAL, and balanced the values of arterial blood gases and cytokines. On the CT scans, a significant decrease in the Hounsfield units was observed, which is indicative of an increase in aerated volume of the lung in the treatment group. The echocardiographic parameters did not reveal a significant difference in the treatment group, and compared with the two groups together it was significant. Also, the histopathology demonstrated reduction in the infiltration of inflammatory cells and pulmonary hemorrhage and edema in the recipients of BM-MSCs.

In natural conditions, cells from bone marrow migrated to chemotactic gradients, but the amount of engraftment was low, and use of exogenous stromal cells can be helpful to this mechanism. The exact mechanisms of MSCs’ actions are not precise [25], and sometimes the results of stromal cell research using animal models are incompatible with each other [23]. However, three mechanisms have been defined for MSCs’ actions consisting of differentiation, cell–cell contact and paracrine function via the soluble factors [25, 26]. Researchers are likely to focus on manipulation of inflammatory pathways and optimizing lung repair while preventing ARDS progression [6]; whereas management of inflammatory pathways forbids the development of ARDS, some researchers believe in the immunomodulatory effects of MSCs [6]. Most trials have used local delivery of autologous BM-MSCs with the aim increasing the concentration of growth factors and cytokines in damaged tissue to improve possible engraftment and repair [23]. For the first time, Gupta et al. reported effects of the local delivery of MSCs in ARDS that are consistent with this study [26].

The anti-inflammatory role of MSCs may vary in humans and animals, but the useful effects of MSCs have been shown in several animal models [27] and indicate the significant role of identification feature of MSCs [22]. LPS of gram-negative bacterial wall binds to the CD14/TLR4/MD2 receptor complex, activating pathway and transcription of some inflammation- and apoptosis-related genes and activating innate immune response [18, 25] that produces the acute phase of ARDS. Therefore, the LPS-induced animal models could be suitable for cell therapy. In prior findings, only a few animal models have been used to investigate the mechanism of MSC therapy in ARDS, most of which used rat and mouse [25, 28–31]. Although animal models cannot show all complications of human ARDS, a rabbit can display important physiological and pathological features of human ARDS and can be useful for novel therapeutic strategies [18]. As previously described in this study, we used LPS from *E. coli* strain O55:B5to induce an experimental model of ARDS in the rabbit.

Our results displayed inflammatory cells downregulated by BM-MSCs, which can improve the function of the alveolar-capillary membrane, in agreement with the results of Xiang et al. on improving pulmonary microvascular permeability with MSCs [32]. The reduction of heterophils, macrophages, and the number of total cells were observed in the treated group, unlike the results of other research [26]. The complete blood count findings indicated that despite intrapulmonary administration of LPS and MSCs, there is a relationship between the numbers of intravascular and intra-alveolar heterophils and lymphocytes. Direct administration of LPS effects on gas exchange process and induces acute hypoxemia [33]. The results of studies suggest that MSC transplant can improve hypoxemia via reducing alveolar-arterial oxygen gradient [29, 30] which is in agreement with the present study. But the research results of Moodley et al., (2016) conflicted with this study [27]. The difference in injury model and type of cell therapy may be the cause of the variable results. There are not any studies that have reported clinical signs, including HR, RR, and RT, after stromal cell therapy; this study is the first report in which clinical signs were evaluated using a previously described scoring system.

The protective effects of BM-MSCs on ARDS are a result of the immune regulation function and inhibitory T-cell function [31]. Monocytes and macrophages can release inflammatory factors such as TNF-α that play a role in phagocytes of the necrotic and apoptotic cells [34]. Because MSC soluble factors may be the therapeutic basis of MSCs, this result is confirmed by recent reports that demonstrated immunomodulatory properties of MSCs [26]. MSCs’ effects are explained by a shift from a proinflammatory to an anti-inflammatory response [26]. Reduced cytokines of proinflammatory (TNF-α and IL-6) and increased cytokines of anti-inflammatory (IL-10) in BAL and plasma samples play principal roles in the treatment mechanism of ARDS [26, 29, 30]. In the present study, the TNF-α concentration decreased following MSC therapy, which is similar to many studies [31]. The IL-10 level increased after the use of MSCs and inhibited antigen-presenting cell function and inflammation and enhanced tolerance, but our findings are not similar to some other studies [26, 30]. Beneficial effects of MSCs may be mediated by a decrease of TNF that indicated the attenuation of the inflammation [26]. Prostaglandin E2 (PGE2) has a critical role in IL-10 and IL-6 secretion from macrophages [31]. It is possible that TNF- and IL-6-dependent PGE2 production plays a major role as a clinical sign and in particular hyperthermia in the acute phase of inflammation [34]. TNF has roles in local inflammation complications such as leukocyte stimulation (neutrophils and macrophage) in BAL and lung endothelium that was shown in the pathology. Also, TNF has roles in systemic complications such as hyperthermia, C-reactive protein, leukocytosis, necrosis, and apoptosis, decreasing appetite, and even decreases of cardiac output and vascular permeability and increases in edema and hypotension.

In this study, CT scans and echocardiography were used to follow improvement and management of ARDS during BM-MSC therapy. One particular feature of ARDS is lack of aerated lung volume caused by inflammation and edema, and total lung volume reduction (air + parenchyma) mostly in the lower lobes can explain hypoxemia, low respiratory compliance, and alveolar dead space and increased pulmonary vascular permeability [35, 36]. CT scanning is a common clinical diagnostic tool, a specific, repeatable, and noninvasive technique for diagnosis that until now has rarely been used as a research tool, and there is not any valid data of the distribution region of the existence or absence of air in alveolus during ARDS and especially after cell-based therapy. Our findings based on CT scanning are matched with the diagnosis of pathology and all the other outcomes. Therefore, it can be said that CT scanning is a suitable method for evaluation of the stromal cell therapy effects in pulmonary inflammation and edema, but further studies are needed in this field. Interactions between lung, right ventricle, and pulmonary circulation are critical in ARDS. Serial echocardiographic measurements have a potential clinical diagnosis in the early stage ARDS as a prognostic and therapeutic method. We showed that ARDS causes changes in echocardiographic parameters and reduces cardiac function, whereas transplant of BM-MSCs was able to prevent these changes.

We demonstrated that MSCs could attenuate the inflammation by reducing pathological lung changes [31], but in some research, no significant data were reported regarding pathology. This variation may be caused by short study duration so that further studies are needed. Our histopathological examination, like other findings from imaging and all of the laboratory results, showed that BM-MSC transplant could improve ARDS.

## Conclusions

Many studies on cell-based therapies in ARDS have been done, but most of them focused on molecular and signal tests. Although these studies could clear pathways, they are still far from tissue function. In this study, we tried to explain the MSCs’ effects on organ function. We investigated effects of BM-MSCs in an experimental model of ARDS and confirmed that MSCs decrease inflammation and improve alveolar fluid clearance and have a protective role in ARDS. Improvement in clinical signs, the decrease of inflammatory cells in blood and BAL, the balance in blood gases and cytokines, the decrease in the Hounsfield units, no changes in echocardiographic parameters. and the reduction of pulmonary hemorrhage and edema in pathology were observed. Despite these results, subsequent studies are required to confirm the decrease in inflammation, and physiological parameters over the long term and many experiments should be performed until stromal cell therapy is validated as a method of routine clinical treatment.

## Additional file

Additional file 1: Additional methods, Figures S1–S6 and Tables S1–S7. (DOCX 1420 kb)

## Abbreviations

ABG: Arterial blood gases
ALI: Acute lung injury
AnGap: Anion gap
AO: Aortic root diameter
ARDS: Acute respiratory distress syndrome
BAL: Bronchoalveolar lavage
BM: Bone marrow
BM-MSCs: Bone marrow mesenchymal stem cells
CD14: Cluster of differentiation 14
CT: Computed tomography
’EDV: End-diastolic volume
EF: Ejection fraction
ESV: End-systolic volume
FS: Fractional shortening
GMP: Good manufacturing practice
HCO_3_: Bicarbonate
Hct: Hematocrit
Hgb: Whole-blood hemoglobin concentration
HR: Heart rate
IVSd: Interventricular septal end diastole
IVSs: Interventricular septal end systole
LA: Left atrium diameter
LPS: Lipopolysaccharide
LVID: Left ventricular internal dimension
LVOT_Vmax_: Left ventricular outflow tract maximum velocity
LVOT_Vmean_: Left ventricular outflow tract mean velocity
LVPWd: Left ventricular posterior wall end diastole
LVPWs: Left ventricular posterior wall end systole
MCHC: Mean erythrocyte hemoglobin concentration
MSCs: Mesenchymal stem cells
PCO_2_: Partial pressure of carbon dioxide
PCV: Packed cell volume
Plt: Platelets
PO_2_: Partial pressure of oxygen
RBC: Red blood cell
RR: Respiratory rate
RT: Room temperature
RVOT_Vmax_: Right ventricular outflow tract maximum velocity
RVOT_Vmean_: Right ventricular outflow tract mean velocity
SatO_2_: O_2_ saturation
SV: Stroke volume
TCO_2_: Total CO_2_
TLR4: Toll-like receptor 4
TNF-α: Tumor necrosis factor-α
WBC: White blood cell
