# Supplementary material for: Intrapulmonary autologous transplant of bone marrow-derived mesenchymal stromal cells improves lipopolysaccharide-induced acute respiratory distress syndrome in rabbit
Source: Crit Care. 2018 Dec 20;22:353. doi: 10.1186/s13054-018-2272-x (PMC6302408; doi:10.1186/s13054-018-2272-x)
Supplement: Supplementary file 1 — Additional methods, Figures S1–S6 and Tables S1–S7. (DOCX 1420 kb) [file 13054_2018_2272_MOESM1_ESM.docx]

**Additional Methods**

**Animal Care**

All the male rabbits (weighing 2±0.2 kg) were housed in single cages in appropriate laboratory conditions (temperature of 24±2 °C, 12-h lighting and darkness cycles) in the Institute of Biomedical Research, University of Tehran. Before starting the study, rabbits with signs of systemic disease on clinical examination, abnormalities on the lung pattern in X-ray images, and symptoms of congenital or acquired heart disease in echocardiograms were excluded. All experimental protocols and animals were reviewed and appropriated by the Animal Research Ethical Committee of University of Tehran (Tehran, Iran).

**Isolation, Primary** **culture, and Expansion of BM-MSC**

Ten male, White New Zealand rabbits, were anaesthetized by an intramuscular injection of 35 mg/kg ketamine 10% (Alfasan-Holland), and 5 mg/kg xylazine 2% (Interchemie-Holland) and were given tramadol 10mg/kg (Aburaihan -Iran) as an analgesic. Bone marrow (BM) samples (about 5 ml) were obtained from humerus in the condition of aseptic surgical by 16-G needle and syringe containing heparin (700 u/ml). Then, BM-MSCs were generated under GMP conditions**.**

At first, DMEM-HG (Gibco, USA) was added BM. Then, samples were transferred to 15 ml sterile tubes containing 3 ml of Ficoll-Paque (Biowest-France). After 30 min of centrifugation (400 rcf), MNCs were collected from the interphase (cloud-like cell layer), eventually the cell pellets were seeded into 25 cm^2^ flasks (SPL Life) with DMEM-HG, 20% FBS (Gibco) and 100 U/ml penicillin/streptomycin (Biowest, France) and incubated at 37 °C in humid air with 5% CO2 (Memert, USA). After 72 h of cultivation, BM-MSCs adhered to the culture plates, and the non-adherent cells that remained suspended in the medium were removed from the culture by changing the medium. The medium was replaced every 3–4 days. When the adhesion of the cells was near confluence (more than 70%), they were trypsinised by trypsin-EDTA of 0.25% (Gibco, USA) and re-plated at dilutions of 1:2 under conditions of the same cultivation. Culture was distended to passage 3 and was harvested for use in in vivo experiment. The number of BMSCs at passage 3 was determined with trypan blue staining (5×10^6^ cells per kilogram of recipient body weight).

**Immunophenotypic characterization of BM-MSCs**

The BM-MSCs at passage three were harvested by trypsin, washed with PBS, and labeled with PE-conjugated antibodies against CD45 (Biolegend, Inc), CD90 (eBioscience), CD34 and CD29 (Abcam, Inc). The cells were analyzed using flow cytometry (BD Bioscience, USA) using standard methods and Flowjo version 7.6.1 software.

**Osteogenic and adipogenic differentiation of BM-MSCs in vitro**

BM-MSCs from 3 passages were seeded at an initial concentration of 1×10^5^ cells/well in Coated 6-well tissue culture plate with ten µg/ml bovine fibronectin (promocell, C-43050) and we worked in duplicate. For osteogenic differentiation, after 24h, cells were treated with osteogenic differentiation medium, and 50% of the medium was changed twice a week. After 21 days, the cells were washed with negative PBS, fixed with Paraformaldehyde of 4% for 20 min at RT and stained for 10 min with Alizarin Red (Bioidea-Iran) to expose the sediment of the calcium-rich mineralized matrix. Alizarin Red was removed by washing with distilled water.

Adipogenesis was performed by seeding cells at the conditions stated above. After 24 h, the medium was replaced by adding adipogenic differentiation medium. 50% of the medium was restored twice a week for 14 days. Then, cells were washed with PBS, fixed with 4% Paraformaldehyde for 20 min at RT and stained with Oil Red O (Bioidea-iran) for 15 min at RT, then cells were washed with [distilled water](http://en.wikipedia.org/wiki/Distilled_water) to remove excess stain and observed using the Olympus IX71 inverted microscope.

**Experimental design**

**ARDS experimental model**

Ten healthy adult male New Zealand white rabbits were chosen, and ARDS experimental model was induced. LPS induced direct lung inflammation under general anesthesia with 35 mg/kg ketamine 10% and 5 mg/kg xylazine 2%. Rabbits were treated with LPS from *E. Coli* O55: B5 [[20](#_ENREF_20)] (Sigma-Aldrich) at 400 µg/kg in 0.1 ml PBS intrapulmonary under bronchoscopy guidance, and the animals were allowed to recover. This dose has been confirmed by findings of clinical symptoms, radiography, Chest CT-scan, complete blood count and blood gas analysis from different doses (50, 100, 200, 400, 800 µg/kg). After the ARDS confirmation, rabbits were randomly distributed into two groups: (i) control group (ARDS + PBS): 5 rabbits were injected via the bronchoscope with PBS 24 h after being instilled with LPS intrapulmonary; and (ii) treatment group (ARDS + BM-BMSC): 5 rabbits were injected via the bronchoscope with BM-BMSCs 24 h after being instilled with LPS intrapulmonary.

**BM-MSCs Autologous Transplantation**

24 h after induction of ARDS, the rabbits were sedated by intramuscular injection of ketamine and xylazine. Then, 10^10^ BM-MSCs suspended in 0.1 ml PBS [[5](#_ENREF_5)] were autologous transplanted intrapulmonary under bronchoscopy guidance. Before preparation for infusion, cells viability and number were estimated using trypan blue staining. The animals were monitored after BM-MSCs autologous transplantation. Clinical assessment, BAL analysis, measurement of haematologic parameters, ABG, BAL and plasma cytokines were determined before transplantation and then for 3,6,12,24,48,72,168 h after transplantation.

**Analyses**

**Clinical assessment**

During the study, the clinical signs of rabbits were calculated and recorded based on clinical scores for each rabbit. HR, RR, body temperature*,* twitch, abnormal breathing, nasal discharge, cough, appetite, physical condition were measured using a clinical score. The scoring is based on clinical evaluated criteria that were individually defined and measured for each rabbit (Table1).

**Imaging**

**Radiography**

Radiographic examination of the thorax was performed for intial evaluation of the intrathoracic structures. Two standard thoracic radiographic positions, Lateral and Ventro-dorsal, were performed by digital X-ray Kodak carestream directview classic CR (Toshiba) equipment, the techniqal factors were –Kv and –mA for every individual. The rabits with radiographic evidences of cardiovascular and pulmonary involvement were excluded from the study.

**Computerized Tomography Scan (CT-Scan)**

The thoracic CT-scan was performed by the aid of Somatoma Spirit class II (Siemens) equipment of Small animal hospital of Tehran University. For CT-scan examination each rabbit was sedated by intramuscular injection of ketamine (35 mg/kg) and xylazine (5 mg/kg) cocktail. The animals were positioned in sternal recumbency and head toward the gantry. The plain CT scan was performed with technical factors of 130 Kv, 30 mA, rotation time: 0.8, slice thickness of 1mm and pitch: 0.5, from apical thoracic inlet until L2. All acquired images were reconstructed into pulmonary and soft tissue windows. The CT-scan examination was performed during experimental modeling of ARDS, before BM-MSCs transplantation and repeated 12, 24, 48, 72 and 168 h after transplantation in each animal under the same circumstances.

**Echocardiography**

Echocardiographic examinations were performed using a GE ultrasound unit Vivid 7 model (Norway) with a 4.4-10.0 MHz phased-array transducer (10S). The rabbits were restrained on the right lateral recumbency with extended forelimbs. All rabbits were tested by two-dimensional, M-mode and Pulsed-wave Doppler echocardiography and were examined with right parasternal trans-thoracic echocardiography for physical, cardiologic and conventional hematological disorders. Echocardiography measurements were recorded at baseline and were repeated at a different time during the experiment (12, 24, 48, 72 and 168 h) to evaluate cardiac function before and after stem**/**stromal cells transplantation. Trans-thoracic echocardiography examination was performed to determine SV, LVID, LVPWs, LVPWd, IVSs, IVSd, FS, EF, ESV, EDV, RVOT _Vmax_, LVOT _Vmax_, RVOT _Vmean,_ LVOT _Vmean_, LA, AO and LA/AO.

**Sampling**

**Blood samples**

Two blood samples were collected from the central ear artery and vein of rabbits into 1-mL syringes containing the anticoagulation before BM-MSCs transplantation and then for 3, 6, 12, 24, 48, 72 and 168 h after transplantation. Arterial samples containing heparin were used for blood gases analyses, and the venous samples containing EDTA were distributed in two microtubes. One micro tube was immediately used for haematologic parameters analysis, and the other was centrifuged at 805 g at 4 °C for 20 min. Then, plasma was separated and frozen at -80 °C for measurement of cytokines.

**BAL samples**

After general anaesthetizing of rabbits with ketamine and xylazine, they were sternaly placed, and fiberoptic bronchoscope (KARL STORZ- 11262BC) was guided through the mouth until carina for the collection of BAL samples before BM-MSCs transplantation and then 24, 48, 72 and 168 h after transplantation. 1-2 ml sterile normal saline/kg (room temperature) was instilled into the carina through the biopsy channel of the bronchoscope and immediately aspirated. Then the BAL samples were collected in 1 ml tube and centrifuged at 400 g at 4 °C for 10 min, after this, pellets were used for complete cell counts and the supernatants were stored at -80 °C for measurement of cytokines.

**Haematologic parameters analysis**

Complete haemogram was determined in venous blood samples by standard methods. Hematological parameters including PCV/HCT, HGB, WBC, RBC, MCHC, granulocyte %, lymphocyte + monocyte count, lymphocyte/monocyte % and platelet count (PLT) were measured by automated blood cell counter (Nihon Kohden). The separation of leukocyte amount was carried out on routinely prepared Geimsa-stained blood lams by cross-sectional technique.

**Blood gases analysis**

Blood gases analysis was immediately performed after sampling on arterial blood samples by Blood Gas Analyzers (OPTI CCA-TS). These parameters include pH, PO2, PCO2, HCO3, TCO2, SatO2 and AnGap and electrolytes such as Na^+^, K^+,^ and Cl^-^.

**Measurement of cytokines in plasma and BAL**

For this purpose, plasma and BAL samples were removed from the freezer at -80 ⁰C. The cytokines concentrations of pro and anti-inflammatory such as TNF-α, IL-6, and IL-10 were measured with a commercially available ELISA kit (Eastbiopharm-USA) following the manufacturer's protocols.

**Determination of Complete Cell Counts in BAL**

Smears were prepared from pellets obtained from centrifugation of the BAL samples. Then, smears were stained using Giemsa; Total cell count, macrophage and heterophiles were counted from the total 100 cells.

**Histopathology**

The rabbits were sacrificed seven days after BM-MSC transplantation using an intravenous injection of 100 mg/kg thiopental sodium. Then, the thoracic cavity was cut, and the lungs and hearts were ligatured, dissected and removed from the chest. First, the lungs and hearts were macroscopically examined, and abnormalities were recorded. Then the tissue sections of the lungs and hearts were fixed in neutral-buffered formalin of 10%, and the process was routinely done, dehydrated and embedded in paraffin wax, cut into thickness of 5 µm (Rotary Microtome RM2145; Leica) and stained with H&E and seen by Nikon Optical Microscope (E600 Eclipse, Japan). Photographs from tissue samples were prepared using a digital camera (u eye 2250) with a microbial software version 2. Finally, the images were processed using AxioVision software, version 4.8.

**Additional figures**

**
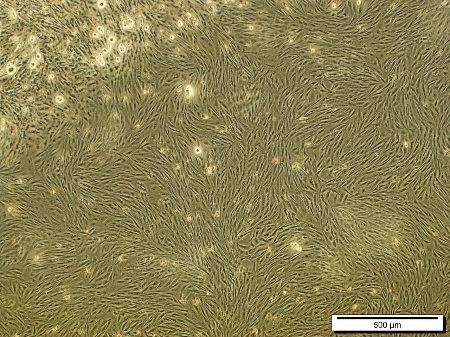
**

Figure S1: The microscopic spindle-shaped and fibroblast-like morphology of BM-MSC at passage 3, Bars: 500 µm


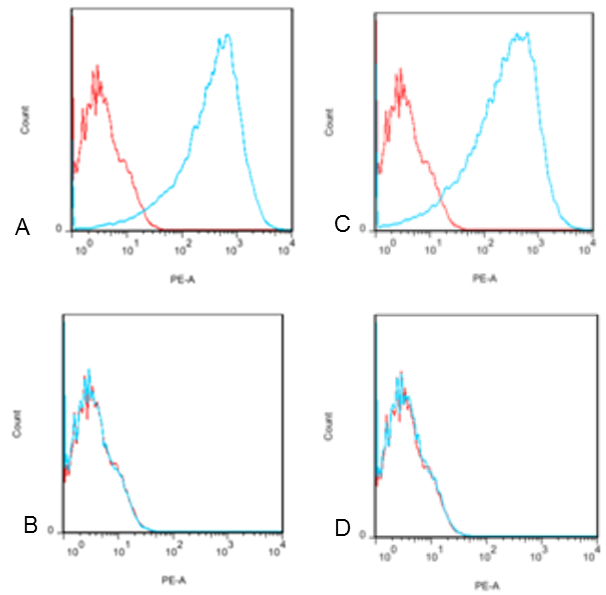


Figure S2: **Flow cytometric analysis of BM-MSCs.** The red lines displayed the nonstained control, and the blue lines showed the level of the surface markers. Flow cytometry analysis demon­strated expression of cell surface markers of CD29 (92%) (A) and CD90 (89%) (B) and were negative for CD34 (C) and CD45 (D).


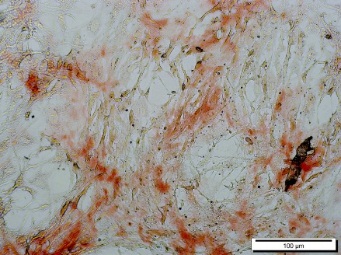

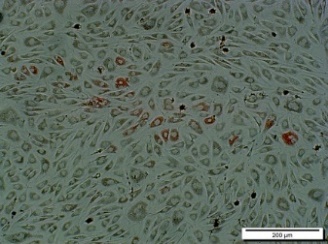


**A**

**B**

Figure S3: **Differentiation of BM-MSCs into osteocytes and adipocytes.** Multi lineage differentiation ability of BM-MSCs to osteogenic and adipogenic in vitro confirmed potential pluripotent MSCs. (A) Osteocytes were differentiated with alizarin red staining of passage three BM-MSCs. Bars: 100µm, (B) Adipocytes were differentiated with red oil staining of passage three BM-MSCs. Bars: 200 µm


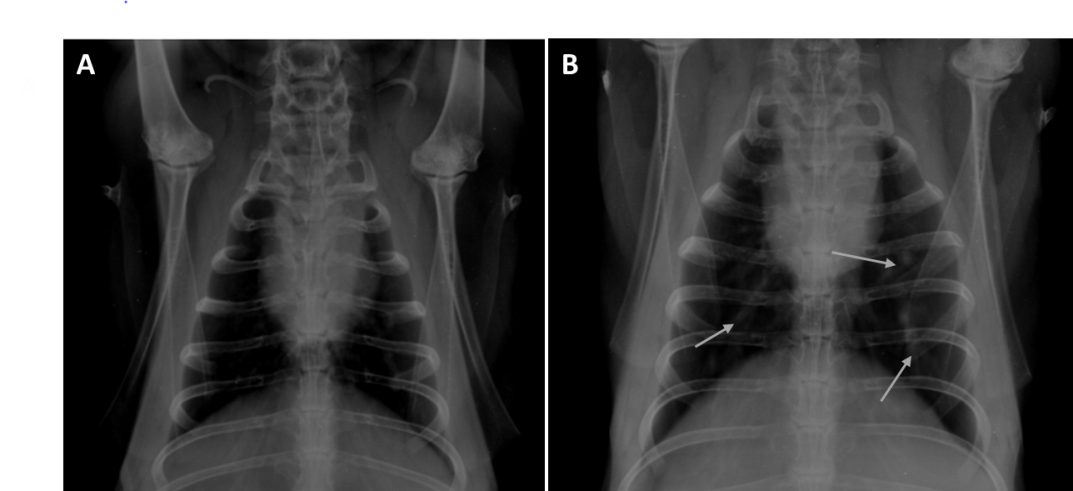


Figure S4: **Radiograph of the chest in the rabbit.** (A) The normal lung, (B) Ventrodorsal view of the inflammated lung. The alveolar pattern is noted (arrows).

**
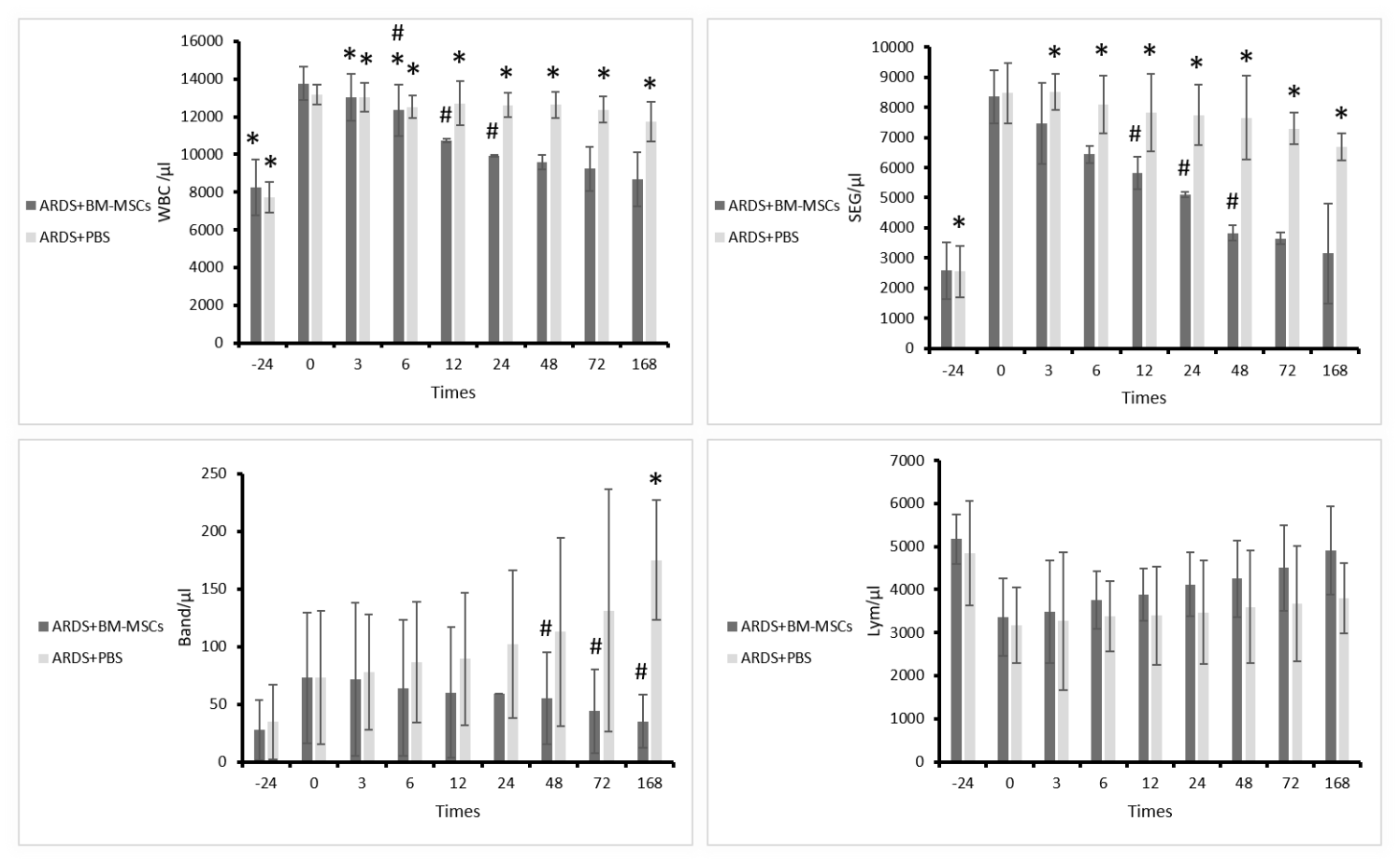
**

Figure S5: The hematological parameters of rabbits (mean± SD) in the groups of treatment (ARDS+BM-MSCs) and control (ARDS+PBS) during the different times of sampling. (A) WBCs, (B) Segmented heterophils, (C) Band heterophils, (D) lymphocytes.

| 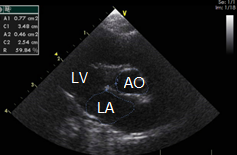  A | 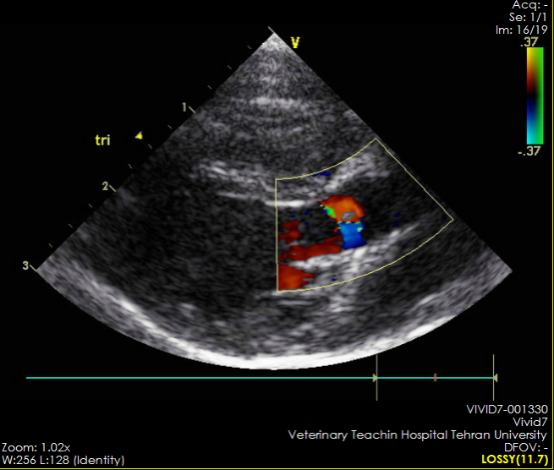  **B** |  |
| --- | --- | --- |

Figure S6: **Echocardiograms in parasternal long axis view in the rabbit.** (A) Apical five-chamber view: five chambers include the right (RV) and the left ventricle (LV), as well as the aortic bulb (AO) and the right (RA) and left atrium (LA) are observed, and AO and LA were measured using M-mode tracing during diastole. (B) A mild regurgitation was nocied on color flow Doppler echocardiogram. The colorful jet represents the blood leaking backwards into the tricuspid valve.

| Table S1: Scored system of clinical symptoms in the rabbit. | | | | | |
| --- | --- | --- | --- | --- | --- |
| **Score**  **Symptoms** | 0 | 1 | 2 | 3 | 4 |
| **Appetite** | Normal | Decrease (<50%) | Decrease (≥50%) | Anorexie | - |
| **A cough** | Absent | A single cough | Involuntary, repetitive coughs | Repetitive coughs | - |
| **Physical condition** | Normal  (alert) | Partial depression (slowly response) | Moderate depression (acutely slow response) | Severe depression (sleeping) | - |
| **Lung sounds** | Normal | - | - | Abnormal  (wheeze, crackle, friction sound) | - |
| **Twitch** | Normal | - | - | Decrease twitching | - |
| **Nasal discharge** | Absent | Serous | mucus | Mucu-purulent | Purulent |

| Table S2: The vital signs of rabbits (mean± SD) in the treatment (ARDS+BM-MSCs) and control (ARDS+PBS) groups during the different times. | | | | | | | | | | |
| --- | --- | --- | --- | --- | --- | --- | --- | --- | --- | --- |
| **Time (h)**  **Symptoms** | Group | -24 | 0 | 3 | 6 | 12 | 24 | 48 | 72 | 168 |
| **HR**  **(bpm)** | Treatment | 166.4±5.89 | 190±3.53 | 191.6±11.76 | 186.8±14.04 | 182.4±14.25 | 175.8±2.38 | 172.4±10.35 | 173.8±7.62 | 170.4±12.28 |
|  | Control | 166±4.18 | 191.8±3.49 | 192±11.31 | 194.8±8.89 | 195.6±15.38 | 192±21.67 | 193.8±25.53 | 189.2±17.35 | 186.8±10.44 |
| **RR**  **(bpm)** | Treatment | 70±6.16 | 116.6±4.44 | 116.2±4.02 | 112.6±8.20 | 108.2±9.47 | 100.2±3.76 | 96±3.24 | 91.6±2.60 | 83.6±6.80 |
|  | Control | 73.6±5.17 | 117.2±11.62 | 120.4±8.04 | 120±9.69 | 115.2±4.60 | 116.4±6.98 | 115.2±6.87 | 110.8±7.82 | 105.2±2.77 |
| **Temperature**  **(ºc)** | Treatment | 38.72±0.16 | 40.46±0.26 | 40.12±0.61 | 39.44±0.28 | 38.94±0.44 | 38.64±0.37 | 38.7±0.89 | 38.64±0.39 | 38.66±0.35 |
|  | Control | 38.74±0.19 | 40.28±0.32 | 40.56±0.36 | 40.46±0.28 | 40.24±0.20 | 40.12±0.19 | 40.18±0.31 | 40.14±0.30 | 40.04±0.20 |

| Table S3: The number of BAL cells of rabbits (mean± SD) in the treatment (ARDS+BM-MSCs) and control (ARDS+PBS) groups during the different times of sampling. | | | | | | | | |
| --- | --- | --- | --- | --- | --- | --- | --- | --- |
| **Time (h)**  **Cells** | Group | -24 | 0 | 12 | 24 | 48 | 72 | 168 |
| **Macrophage**  **(µl)** | Treatment | 349.6±132.86 | 449.2±119.32 | 440±113.93 | 424.4±105.87 | 441±100.86 | 399.8±115.93 | 386±115.40 |
|  | Control | 350.8±145.18 | 442.6±143.89 | 453±137.26 | 446±135.06 | 443.2±122.50 | 433.6±129.64 | 420.8±120.49 |
| **Heterophile**  **(µl)** | Treatment | *7.8±1.30 | 85.8±4.26 | *65.6±7.02 | *51±7.38 | #*40±2.54 | #*33.6±2.79 | #*24.4±2.50 |
|  | Control | *7.2±1.30 | 86.6±4.72 | *90.2±7.25 | *93.2±7.79 | *88.6±14.99 | *80.8±12.75 | *72.8±13.08 |
| **total cell**  **(µl)** | Treatment | *427.4±136.60 | 605±117.06 | 575.6±115 | #545.4±99.67 | #521±97.30 | #499.4±92.06 | 480.4±88.12 |
|  | Control | *428±146.77 | 599.2±144.36 | *613.2±135.37 | *607.2±178.46 | *603.8±161.44 | *584.4±166.33 | *563.6±133.93 |

| Table S4: The number of arterial blood gases of rabbits (mean± SD) in the treatment (ARDS+BM-MSCs) and control (ARDS+PBS) group during the different times of sampling. | | | | | | | | | | |
| --- | --- | --- | --- | --- | --- | --- | --- | --- | --- | --- |
| **Time (h)**  **Blood gases** | Group | -24 | 0 | 3 | 6 | 12 | 24 | 48 | 72 | 168 |
| **PO_2_**  **(mmHg)** | Treatment | *82.2±4.32 | #58.6±3.36 | 69±12.4 | 69.6±8.84 | #*76±3.08 | #*82.4±0.54 | #*82±1 | 79.4±6.18 | 77.8±4.96 |
|  | Control | *77.8±4.81 | 57.4±1.14 | 64.8±7.25 | 64.2±7.04 | 63.6±10.06 | 64.6±6.22 | 61±2.44 | 64±9.46 | 63.6±8.29 |
| **SaO_2_**  **(%)** | Treatment | *94.8±1.30 | 87.2±1.92 | 89.4±3.43 | #91.4±2.96 | 93.2±1.30 | #94.2±0.83 | #94.2±0.83 | #94.4±0.54 | 93.4±1.94 |
|  | Control | *93.8±1.78 | 87.2±1.92 | *87.8±1.92 | *87.6±0.89 | 88.2±2.58 | 88.4±3.64 | 88.8±4.14 | 88.6±4.50 | 89±3.67 |
| **PCO_2_**  **(mmHg)** | Treatment | *29.4±5.50 | 40.8±3.56 | 39.6±6.50 | 38.8±7.59 | 34.4±5.94 | 32±2.23 | #30.8±2.48 | #30.6±2.07 | 30.4±3.20 |
|  | Control | *29.6±2.40 | 38.8±1.78 | 38.4±4.61 | 37.4±4.82 | 36.6±5.81 | 35.8±6.45 | 33.8±7.46 | 32.8±5.44 | 32.4±2.88 |
| **PH** | Treatment | 7.44±0.08 | 7.34±0.07 | 7.31±0.11 | 7.3±0.12 | 7.33±0.12 | #7.41±0.08 | 7.39±0.14 | 7.39±0.13 | 7.38±0.19 |
|  | Control | 7.42±0.09 | 7.28±0.04 | 7.29±0.19 | 7.28±0.20 | 7.25±0.20 | 7.25±0.25 | 7.25±0.45 | 7.26±0.22 | 7.3±0.13 |
| **HCO_3_**  **(mmol/l)** | Treatment | 21.76±0.80 | 20.38±1.28 | 20.34±0.81 | 20.36±1.40 | 20.52±1.41 | 20.72±1.31 | 20.86±1.65 | 21.06±1.82 | 21.12±1.43 |
|  | Control | 22.04±2 | 20.52±1.66 | 20.42±1.55 | 20.36±1.85 | 20.32±1.80 | 20.36±2.47 | 20.66±2.62 | 20.82±2.09 | 20.9±1.85 |
| **BE**  **(mmol/l)** | Treatment | 1.12±1.23 | 1.22±1.19 | 1.28±1.24 | 1.26±1.31 | 1.24±1.28 | 1.24±1.33 | 1.2±1.33 | 1.18±1.25 | 1.16±1.27 |
|  | Control | 0.92±1.69 | 1.04±1.67 | 1.08±1.78 | 1.1±1.77 | 1.2±1.8 | 1.26±1.84 | 1.24±1.78 | 1.22±1.79 | 1.18±1.79 |
| **Anion Gap**  **(mmol/l)** | Treatment | 15.24±0.89 | 15.3±1.63 | 15.36±2.76 | 15.4±2.63 | 15.34±2.55 | 15.3±2.69 | 15.32±3.26 | 15.3±2.77 | 15.28±2.80 |
|  | Control | 15.34±1.61 | 15.38±1.50 | 15.46±1.56 | 15.52±1.69 | 15.56±1.62 | 15.58±1.81 | 15.56±2.84 | 15.54±2.96 | 15.5±2.43 |

| Table S5: The amount of arterial blood electrolytes of rabbits (mean± SD) in the treatment (ARDS+BM-MSCs) and control (ARDS+PBS) groups during the different times of sampling. | | | | | | | | | | |
| --- | --- | --- | --- | --- | --- | --- | --- | --- | --- | --- |
| **Time (h)**  **Electrolytes** | Group | -24 | 0 | 3 | 6 | 12 | 24 | 48 | 72 | 168 |
| **Na**  **(mmol/l)** | Treatment | 140.6±6.10 | 142.8±2.16 | 145.8±4.38 | 145±3 | 142.8±4.14 | 143.6±1.34 | 143.2±3.49 | 141.8±2.68 | 141.4±2.88 |
|  | Control | 140.4±1.14 | 142±3.53 | 145.6±1.14 | 143±4.18 | 143.4±2.96 | 142.8±1.78 | 143±1.41 | 142.8±2.16 | 141.6±1.81 |
| **K**  **(mmol/l)** | Treatment | 3.6±0.51 | 3.3±0.17 | 3.24±0.42 | 3.28±0.22 | 3.32±0.32 | 3.32±0.26 | 3.34±0.37 | 3.38±0.27 | 3.46±0.48 |
|  | Control | 3.5±0.70 | 3.2±0.71 | 3.14±0.61 | 3.16±0.47 | 3.26±0.25 | 3.3±0.50 | 3.28±0.49 | 3.3±0.40 | 3.4±0.33 |
| **CL**  **(mmol/l)** | Treatment | 110.2±1.48 | 111±1.22 | 112±4.30 | 112.6±3.04 | #111.8±3.49 | 111.2±0.83 | 111.4±1.34 | 111±2.12 | 110.6±1.34 |
|  | Control | 109.4±2.96 | 110.4±2.70 | 111.2±2.38 | 111.6±2.40 | 112.4±1.67 | 111.8±1.78 | 111.2±0.83 | 110.6±0.89 | 110.8±0.83 |

| Table S6: The amount of total protein of BAL and BAL and plasma cytokines of rabbits (mean± SD) in the treatment (ARDS+BM-MSCs) and control (ARDS+PBS) group during the different times of sampling. | | | | | | | | |
| --- | --- | --- | --- | --- | --- | --- | --- | --- |
| **Time (h)** | Group | -24 | 0 | 12 | 24 | 48 | 72 | 168 |
| **Total pr**  **(µg/ml)** | Treatment | *229.63±25.65 | 279.11±35.04 | 265.12±30.60 | #261.79±19.15 | #248.42±14.93 | 242.8±18.22 | #235.44±14.20 |
|  | Control | *229.88±21.09 | 278.21±28.34 | 281.04±43.82 | 279.05±40.59 | 273.17±43.46 | 265.45±30.02 | 260.19±28 |
| **TNF-α BAL**  **(pg/ µg concentrated pr)** | Treatment | 0.098±0.008 | 0.106±0.011 | #0.957±0.009 | 0.923±0.0086 | #0.092±0.004 | 0.095±0.007 | 0.097±0.006 |
|  | Control | 0.1±0.013 | 0.108±0.013 | 0.102±0.015 | 0.101±0.016 | 0.102±0.019 | 0.104±0.021 | 0.104±0.023 |
| **TNF-α Plasma**  **(pg/mL)** | Treatment | *15.58±0.56 | 24±0.65 | 20.52±2.76 | 19±1.14 | 18.08±0.84 | #17.72±0.53 | #17.48±0.49 |
|  | Control | *15.6±0.91 | 24.26±0.77 | *23.72±1.71 | *22.66±0.93 | 22.44±1.99 | 21.96±2.11 | 20.82±1.27 |
| **IL6 BAL**  **(pg/ µg concentrated pr)** | Treatment | 0.92±0.20 | 1.22±0.11 | *#0.97±0.11 | *#0.92±0.06 | *0.95±0.06 | 0.95±0.17 | #0.95±0.10 |
|  | Control | 1.02±0.17 | 1.34±0.14 | 1.32±0.43 | 1.28±0.28 | 1.29±0.60 | 1.27±0.36 | 1.22±0.52 |
| **IL6 Plasma**  **(pg/mL)** | Treatment | *109.54±16.64 | 189.3±4.01 | 178.84±19.27 | #161.56±5.25 | #155.82±14.01 | #147.26±15.01 | #129.28±5.33 |
|  | Control | *111.12±13.26 | 189.1±6.17 | *189.2±9.55 | *182.52±16.88 | 171.34±31.41 | 167.68±29.11 | 165.32±20.83 |
| **IL10 BAL**  **(pg/ µg concentrated pr)** | Treatment | *0.81±0.11 | 0.46±0.05 | 0.56±0.10 | *0.59±0.05 | *#0.62±0.03 | 0.63±0.07 | 0.68±0.05 |
|  | Control | *0.78±0.05 | 0.46±0.05 | 0.48±0.10 | 0.47±0.08 | 0.49±0.12 | 0.5±0.12 | 0.54±0.12 |
| **IL10 Plasma**  **(pg/mL)** | Treatment | *150.52±12.37 | 100.46±9.42 | 118.08±21.67 | 118.22±20.32 | #122.22±4.37 | #136.38±18.64 | 143.22±15.25 |
|  | Control | *147.18±17.41 | 98.8±11.52 | *106.58±8.88 | 106.06±10.99 | 110.02±16.62 | 115.58±8.39 | 120.04±8.03 |

| Table S7: The amount of CT-scan volumetry (Mean Hounsfield unit and total volume) of rabbits (mean± SD) in the treatment (ARDS+BM-MSCs) and control (ARDS+PBS) groups during the different times. | | | | | | | | |
| --- | --- | --- | --- | --- | --- | --- | --- | --- |
| **Time (h)**  **Parameters** | Group | -24 | 0 | 12 | 24 | 48 | 72 | 168 |
| **Hounsfield unit** | Treatment | -608.5±16.31 | -545.96±8.94 | -565.48±36.25 | -586.42±23.95 | -596.5±13.99 | #-608.56±19.87 | #-602.04±15.63 |
|  | Control | -601.22±10.74 | -544.32±15.07 | -546.3±41.80 | -547.54±51.44 | *-546.1±12.9 | *-542.46±8.23 | -537.42±46.30 |
| **Total volume** | Treatment | 60.92±5.24 | 70.84±1.95 | 71.36±12.15 | 69.98±13.39 | #67.72±5.86 | #64.88±0.27 | #62.26±1.05 |
|  | Control | 60.56±2.85 | 68.64±3.33 | *73.6±3.68 | *76.04±3.58 | *76.98±3.60 | *79.18±3.86 | *81.38±3.50 |

Note: In the all of the tables, data were presented as mean± SD (n =5 rabbit per group).

*p ≤ 0.05; significant compared with inflammation time in the same group.

#p ≤ 0.05; significant compared with the control group at the same time.
